# Forest Guardians: The Role of Dense Forests and Water Networks in Supporting Lowland Tapir (*Tapirus terrestris*) Populations in the Orinoquia Region of Colombia

**DOI:** 10.1002/ece3.72642

**Published:** 2025-12-12

**Authors:** Ángela Alviz, Karen E. Pérez‐Albarracín, Jorge Salazar‐Bravo, Richard D. Stevens

**Affiliations:** ^1^ Department of Biological Sciences Texas Tech University Lubbock Texas USA; ^2^ Fundación Orinoquia Biodiversa Arauca Colombia; ^3^ Department of Natural Resources Management Texas Tech University Lubbock Texas USA; ^4^ Natural Science Research Laboratory Museum of Texas Tech University Lubbock Texas USA

**Keywords:** climate change, conservation, crops, fragmentation, occupancy, cambio climático, conservación, cultivos, fragmentación, ocupación

## Abstract

The lowland tapir ( *Tapirus terrestris* ) is a megaherbivore integral to ecosystem functioning in South America's tropical landscapes but is increasingly threatened by habitat loss and fragmentation. This study assessed occupancy, detectability, and activity patterns of lowland tapirs across the Orinoquia region of Colombia. We deployed 360 camera traps over 32,000 trap‐days from 2015 to 2023 across nine study areas, evaluating the influence of habitat characteristics and anthropogenic factors on the tapir presence using hierarchical occupancy models. Our results revealed a naïve occupancy of 0.40 and an overall detectability of 0.46. Model‐averaged estimates identified dense forest cover as a critical positive predictor of tapir occupancy (Ψ = 0.58, CI: 0.39–0.64), while proximity to water resources similarly enhanced both occupancy and detectability. Conversely, anthropogenic landscapes such as pastures negatively impacted occupancy probabilities (Ψ = 0.45, CI: 0.40–0.51), whereas crop areas, primarily those used for household consumption, showed a surprising positive association. Taken together, these results indicate higher occupancy in dense forest and near streams, lower occupancy in pastures, no strong positive effect of gallery/riparian belts, and a context‐dependent positive association with smallholder crops. Our findings underscore the importance of dense forests and water bodies for tapir conservation and suggest that effective management strategies must address habitat fragmentation and human–wildlife conflicts. Future conservation efforts should include intensive monitoring, community‐based participatory approaches, and consideration of climate change impacts to ensure the long‐term survival of tapir populations in this rapidly changing landscape.

## Graphical abstract

Our study reveals that lowland tapir ( *Tapirus terrestris* ) occupancy in Colombia's Orinoquia region is strongly influenced by dense forests and proximity to water bodies, while anthropogenic factors such as pastures and road networks negatively impact their presence. Using an extensive 8 year camera trap dataset and hierarchical occupancy models, we demonstrate that habitat fragmentation and land‐use change shape tapir distribution, with certain crop types unexpectedly supporting their persistence. These findings underscore the urgent need for conservation strategies that prioritize habitat connectivity, community‐based management, and climate‐resilient approaches to ensure the long‐term survival of this keystone megaherbivore.

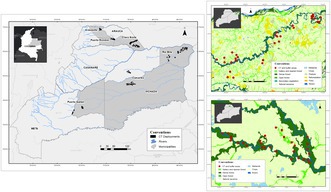

## RESUMEN

La danta de tierras bajas ( *Tapirus terrestris* ) es un megaherbívoro que hace parte integral de la funcionalidad ecológica de los paisajes tropicales de Sur América, el cual se encuentra altamente amenazado como consecuencia de la pérdida y fragmentación de los hábitats. Este estudio buscó determinar la ocupación, detectabilidad y patrones de actividad de la danta de tierras bajas a través de la región de la Orinoquia colombiana. Fueron instaladas 360 cámaras trampa durante 32,000 días efectivos de muestreo entre 2015 y 2023. En total fueron evaluadas nueve áreas de estudio donde se determinó la influencia de las características de hábitat y factores antropogénicos sobre la presencia de las dantas por medio de la implementación de modelos de ocupación jerárquicos. Los resultados revelaron una ocupación naïve de 0.40 y una detectabilidad de 0.46. Las estimaciones promediadas del modelo identificaron el bosque denso como un predictor positivo crítico en la ocupación de la danta (Ψ = 0.58, CI: 0.39–0.64), así como la proximidad a los recursos hídricos mejoró de forma similar tanto la ocupación como la detectabilidad. Por el contrario, los paisajes antropogénicos como los pastos impactaron negativamente en las probabilidades de ocupación (Ψ = 0.45, CI: 0.40–0.51). Las áreas de cultivo, principalmente aquellas utilizadas para el consumo doméstico, mostraron una sorprendente asociación positiva. En conjunto, estos resultados indican una mayor ocupación en bosques densos y zonas cercanas a cuerpos de agua, una menor ocupación en pastizales, la ausencia de un efecto positivo marcado de los bosques de galería o ribereños, y una asociación positiva dependiente del contexto con cultivos de pequeña escala. Las carreteras aparecieron como barreras de dispersión significativas, reduciendo la detectabilidad. Nuestros resultados subrayan la importancia de los bosques densos y los cuerpos de agua para la conservación de la danta y sugieren que las estrategias de gestión efectivas deben abordar la fragmentación de los hábitats, la expansión de las carreteras y los conflictos entre el hombre y la fauna. Los futuros esfuerzos de conservación deberían incluir un seguimiento intensivo, enfoques participativos basados en la comunidad y la consideración de los impactos del cambio climático para asegurar la supervivencia a largo plazo de las poblaciones de dantas en este paisaje rápidamente cambiante.

## 1 Introduction

Anthropogenic disturbances are major drivers of biodiversity loss, significantly affecting both functional and ecosystem diversity (Lacher et al. 2019; Ocampo‐Peñuela et al. 2020; Schaffer‐Smith et al. 2016). Among these disturbances, land‐use change, habitat loss, and fragmentation are some of the greatest threats to biodiversity (Cameron et al. 2022). Habitat fragmentation impacts individual survival, isolates populations, and constrains dispersal to resource‐rich habitat patches (Dertien and Baldwin 2023). These changes compromise ecosystem stability and reduce vital economic and social services for human populations.

Large mammals are particularly vulnerable to fragmentation and other anthropogenic pressures. These species face high extinction risks due to intrinsic factors such as low reproductive rates, sparse populations, and expansive spatial requirements. Fragmentation further exacerbates these risks by limiting population connectivity and dispersal between habitat patches (de la Torre et al. 2018). Globally, about 60% of large herbivores are threatened with extinction (de la Torre et al. 2018; Ripple et al. 2015). Their declines can trigger cascading ecological effects, disrupting ecosystem stability and diminishing services that directly support human populations (Bardavid et al. 2024).

The lowland tapir ( *Tapirus terrestris* ), the largest extant herbivore found in South America, demonstrates the vulnerabilities faced by large mammals in fragmented landscapes. With a long gestation period (approximately 13 months) and low reproductive rates, usually a single offspring per pregnancy (Medici 2010), the species has a limited ability to recover from population declines. Female tapirs reach sexual maturity between 3 and 5 years of age and enter estrus approximately 2–3 months after giving birth, although successful conception may take longer (Padilla and Dowler 1994; Medici 2010). This extended reproductive cycle, combined with habitat fragmentation and increasing anthropogenic pressures, further heightens their susceptibility to local extinction (Varela et al. 2019).

Tapirs are closely associated with forest ecosystems and water bodies, which provide essential resources for refuge, feeding, and courtship (Oliveira‐Santos et al. 2010; Padilla and Dowler 1994). However, the rapid expansion of agriculture and associated land‐use changes has drastically altered the tapir's historical habitat, increasing forest loss and fragmentation (Cordeiro et al. 2016). These pressures are compounded by human activities such as poaching, road construction, and wildfires, which further isolate populations and reduce their resilience to environmental changes (De Paula et al. 2022).

The occupancy of a species, defined as the probability of its presence in a specific site, is a critical indicator of population trends (Mackenzie et al. 2004). For wide‐ranging, elusive species like the lowland tapir, occupancy modeling provides a robust framework to assess the relationships between habitat conditions and species presence, while accounting for imperfect detection (Mackenzie et al. 2005; Schank et al. 2019). Camera trapping, a noninvasive survey technique, is particularly effective in monitoring medium and large mammals (Jansen et al. 2014). It supports occupancy estimation by identifying environmental and anthropogenic influences on species distributions and by examining activity patterns (Bison et al. 2024; Mena et al. 2020).

Understanding species–habitat relationships is crucial for informing effective management and conservation strategies (Betts et al. 2014; Hanski et al. 2013), particularly in biodiversity‐rich but threatened regions like Colombia's Orinoquia. Species like the lowland tapir, whose occupancy rises in dense, riparian forest and declines in monocrops (e.g., rice, oil palm) and pasture mosaics, can serve as candidate indicators of dense and riparian forest integrity and connectivity (i.e., higher canopy cover, proximity to streams, and lower agricultural conversion) (Ferreguetti et al. 2017; Quintero et al. 2023). However, spatial data on the tapir's distribution remain limited, hindering efforts to evaluate population status and habitat use.

This study aimed to address these knowledge gaps by identifying the factors that influence tapir occupancy across landscapes in the Orinoquia region of Colombia. Specifically, we examine the effects of habitat preferences, anthropogenic disturbance, and unique landscape characteristics on both occupancy and detectability. Understanding these dynamics is particularly relevant because the Orinoquia is undergoing rapid land‐use change yet remains critically understudied compared to other South American biomes such as the Atlantic Forest and the Pantanal (Ferreguetti et al. 2017; Regolin et al. 2021). By combining multi‐site, long‐term camera‐trap data and occupancy models that explicitly account for imperfect detection (Mackenzie et al. 2005; Schank et al. 2019), our work provides a robust framework to evaluate how habitat quality, human disturbance, and landscape configuration shape tapir occurrence (Betts et al. 2014; Hanski et al. 2013). These insights establish an empirical foundation for anticipating how this threatened megaherbivore will persist in heterogeneous and changing environments.

## 2 Methods

### 2.1 Study Area

The Orinoquia region of Colombia, located east of the Andes, is a tropical humid biome with warm temperatures ranging from 22°C to 36°C and an average annual rainfall of approximately 2000 mm. This region experiences distinct wet and dry seasons, with the wet season occurring from May to October and the dry season from November to April. These seasonal dynamics influence habitat conditions, with the availability of water resources and vegetation cover fluctuating throughout the year, potentially affecting species distribution and detectability.

The landscape of Orinoquia is characterized by vast savannas interspersed with networks of gallery and riparian forests. These habitats play a critical ecological role as biological corridors, supporting a wide range of biodiversity (Alviz and Pérez‐Albarracín 2019; Correa‐Gómez and Stevenson 2010; de la Viloria Hoz 2009). Unique features such as crescent‐shaped sand dunes (médanos), flowing water masses (raudales), and plant‐dominated areas such as morichales (dominated by *Mauritia flexuosa* ) and congriales (dominated by *Acosmium nitens*) contribute to the region's ecological complexity (de la Viloria Hoz 2009). Savannas are further categorized into woody, open‐seasonal, hyper‐seasonal, and semi‐seasonal types, with hyper‐seasonal savannas being unique to the Llanos regions of Colombia and Venezuela (Minorta‐Cely et al. 2024). Together, these ecosystems regulate water cycles, maintain soil fertility, support plant regeneration through fire–flood dynamics, and provide key food and movement resources for large mammals (Alviz and Pérez‐Albarracín 2019). These ecological processes are essential for maintaining biodiversity and sustaining species such as the lowland tapir, which depends on both forest refuges and open savanna corridors for survival.

In addition to these natural features, the Orinoquia landscape is increasingly influenced by land‐use change. Large areas of savanna and forest are fragmented by cattle pastures, rice fields, and expanding oil palm plantations, especially in Casanare and Meta (Etter et al. 2011; Peñuela et al. 2019; Rincon‐Parra et al. 2022). Road expansion and scattered rural settlements further subdivide habitat, isolating forest patches from one another and reducing connectivity (Blackburn et al. 2021). In contrast, more continuous tracts of dense forest remain along major rivers in areas such as Puerto Rondón (Arauca) and Puerto Gaitán (Meta), where riparian corridors still support relatively intact habitat networks.

### 2.2 Camera Trap Surveys

**FIGURE 1:**
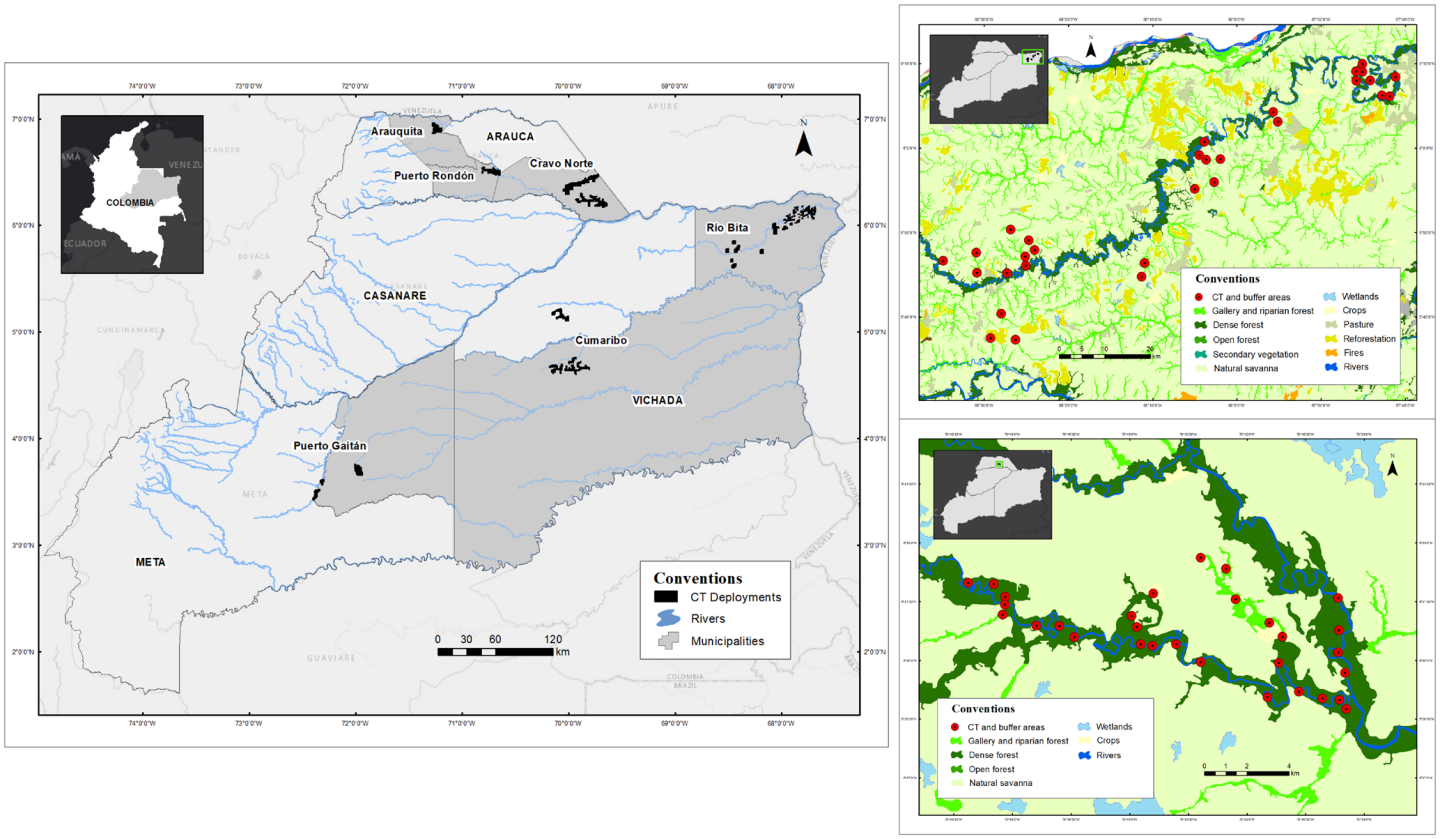
Study area in the Orinoquia region of Colombia showing the nine‐camera trap (CT) deployments used to estimate occupancy and detectability of the lowland tapir. The left panel highlights the general location of all deployments across Arauca, Casanare, Meta, and Vichada. The right panels show detailed land‐cover maps for two representative sites: (top) Puerto Rondón (Arauca) and (bottom) Río Bita (Vichada). Red circles indicate CT stations, each surrounded by a 1‐km buffer.

Nine sampling events were conducted between 2015 and 2023; a total of 360 camera traps were deployed (Bushnell Trophy Cam HD, Trophy Cam HD Aggressor, Core DS No Glow, Core 24 MB Trail Cameras) across nine areas in the four departments of the Orinoquia region (Figure 1). In this study, we define a sampling event as a discrete field campaign in a focal area (e.g., one grid of cameras deployed during a ~3‐month period). Within each sampling event, each camera operated for a contiguous sampling period of 60 trap‐days, which we treated as the temporal unit for assuming demographic closure in the single‐season occupancy model. Each sampling event used a grid system of 1 × 1 km cells with one camera station per cell (target inter‐station spacing ≈1 km center‐to‐center) based on the minimum home range reported for tapirs (Medici et al. 2022). For analyses, we summarized habitat covariates within a 1 km radius circular buffer around each camera (analytical buffer; not a 1 km block containing multiple cameras) to reflect the average daily movement radius of lowland tapirs (~2–4 km^2^ home ranges), ensuring spatial relevance to resource use. Camera traps were installed at 40 sites per area for 60 trap‐nights each. This setup aimed to achieve spatial coverage of diverse habitat types while reducing potential biases. We placed camera traps within each grid cell based on signs of tapir activity (tracks, feeding sites such as palm aggregations, trails, feces) when available; otherwise, cameras were positioned along possible travel routes (e.g., game trails, stream crossings), and a camera was deployed in every selected cell regardless of sign presence. We aimed to sample different habitat types (e.g., dense forest, gallery forest, savannas) across the study area, with sites within each department randomly distributed. Each camera trap operated 24 h per day for the entire deployment period (60 consecutive trap‐nights per station), capturing three consecutive photos per detection with a 1‐s interval between shots. This configuration increased the likelihood of identifying individuals or groups while ensuring high‐quality detection data.

### 2.3 Landscape Covariates

We selected site covariates that potentially affect lowland tapir occupancy and detection based on the available information on its habitat use and natural history (Alviz et al. 2023; Cordeiro et al. 2016; Oliveira‐Santos et al. 2010; Salas 1996; Varela et al. 2019). Covariates were grouped as natural landcover (i.e., type of forest, water), land use change (i.e., crops), and distance (i.e., distance to roads) covariates (Table 1). Forest types and land‐use classes were obtained from the Corine Land Cover Methodology adapted for Colombia (IDEAM 2010), which provides standardized and high‐resolution land‐use data for ecological assessments. The Corine dataset itself is derived from Landsat imagery from 2007–2019 and was updated in 2020, with products available at a scale of 1:100,000. Wildfires were extracted separately through supervised classification of Landsat imagery, identifying burned areas as dark patches contrasting with vegetation, which were digitized into polygons and quantified within the 1 km buffer of each camera station. For each camera station, we intersected the 1 km radius buffer with the Corine Land Cover map and computed the proportion (percent cover) of every class inside the buffer (e.g., dense forest %, gallery forest %, savanna %, crops %, pasture %). Buffers commonly contained multiple habitat types; we retained all as continuous covariates rather than forcing a single dominant type. Hydrology and road layers were used to calculate distance to streams and distance to roads.

**TABLE 1:** Covariates selected to estimate occupancy and detectability probabilities in the Orinoquia region of Colombia.

Type	Variables	ID	Description	Units	Arauca	Casanare	Meta	Vichada
Landcover	Gallery and riparian forest	forest_g	Narrow strip of forest strongly associated with streams.	ha	✓	✓	✓	✓
	Dense forest	forest_d	A forest type characterized by high canopy cover, supporting a complex structure with layers of understory vegetation.	ha	✓	✓	✓	✓
	Open forest	forest_a	Forest with less canopy cover compared to dense forest.	ha	✓			
	Secondary vegetation	sec_veg	Vegetation that regrows naturally or through reforestation after primary forest disturbance, such as logging or fire	ha	✓	✓		✓
	Natural savanna	savanna	An open, grassy ecosystem with scattered trees and shrubs, typically adapted to seasonal dry periods and fire.	ha	✓	✓	✓	✓
	Wetlands	water	Water ecosystems, either seasonally or permanently, including swamps, marshes, and bogs.	ha	✓	✓	✓	✓
Land use change	Crops	crops	Areas used for small‐ or medium‐scale cultivation, including subsistence crops (e.g., plantain, yucca, maize).	ha	✓	✓	✓	✓
	Pasture	pasture	Land primarily used for grazing livestock, often dominated by grasses and sometimes scattered trees.	ha	✓	✓	✓	✓
	Reforestation	refor	Areas replanted with non‐native tree species (e.g., pine, eucalyptus) for timber or other commercial products. These monocultures generally lack structural and species diversity.	ha				✓
	Fires	fires	Uncontrolled fires occurring in natural or semi‐natural landscapes, resulting from human activity.	ha	✓	✓		
Distance	Distance to water	d_water	Euclidean distance from the camera trap to the nearest water body.	m	✓	✓	✓	✓
	Distance to roads	d_roads	Euclidean distance from the camera trap to the nearest road.	m	✓	✓	✓	✓
	Distance to crops	d_crops	Euclidean distance from the camera trap to the nearest crop.	m	✓	✓	✓	✓
	Distance to protected areas	d_protected	Euclidean distance from the camera trap to the nearest protected area.	m		✓		✓

Values for each covariate were first extracted as proportions of land‐cover or land‐use classes within the 1 km buffer around each camera station (e.g., % dense forest, % pasture, % crops). Continuous variables such as distances to roads and streams were also extracted at this buffer scale. These raw values were then standardized (z‐scores: subtracting the mean and dividing by the standard deviation) to ensure comparability of effect sizes across covariates in the occupancy models (Zuur et al. 2010). To address multicollinearity and reduce redundancy, we calculated Pearson's pairwise correlation coefficients among covariates and removed those with an absolute correlation coefficient (|*r*|) greater than 0.7 (Betts et al. 2014; Fiske and Chandler 2011). This threshold balances the retention of ecologically significant variables while minimizing collinearity issues that could mask covariate effects (Dormann et al. 2013). All raster data processing was conducted using ArcGIS Pro 3.2, and subsequent statistical analyses were implemented in R version 4.2.3. This approach integrated spatial and statistical tools to ensure flexible handling of landscape data within the modeling framework.

### 2.4 Occupancy Modeling

We employed a hierarchical modeling approach to account for imperfect detection in our occupancy analysis. Detection histories were generated from camera trap photographs and organized into 4‐day sampling intervals. This approach minimized the presence of zeroes in the dataset, thereby producing a more consistent detection history and reducing data heterogeneity. Detection matrices were constructed as binary records (1 = detection, 0 = non‐detection) following the methods of MacKenzie et al. (2006).

A single‐season occupancy model was implemented separately for each study area, incorporating relevant site covariates (MacKenzie et al. 2006; Niedballa et al. 2015). Model fit was assessed using the *unmarked* package in R (Fiske and Chandler 2011), where all selected covariates were evaluated for their influence on both occupancy and detection probabilities. Our primary objective was to examine tapir occupancy responses to covariates related to land cover and land use at each site. The occupancy model used in this study is based on several key assumptions: (1) Demographic closure within the study area during the sampling period, meaning that there is no immigration, emigration, or local extinction at any site; (2) No false detections, indicating that any detection accurately reflects species presence; and (3) Independence of detection events across spatially independent sites, which means that detection at one site does not influence detection at other sites (Rota et al. 2009). Given the low reproductive rate of tapirs, we assumed local populations remained closed during our sampling period and applied a closed, single‐season occupancy model with a static occupancy probability (Ψ) (Bailey et al. 2007). While subadult lowland tapirs have been reported to disperse in search of suitable habitats or mating partners, recent studies suggest that their movement patterns do not significantly differ from those of adults (Medici et al. 2022). Home‐range sizes and movement speeds were similar between age groups, with subadults exhibiting comparable space use to adults. This suggests that, although dispersal may occur, it is not a frequent or large‐scale event that would strongly violate the demographic closure assumption within our study period.

To construct a robust model set for each study area, we utilized parallel processing to fit multiple occupancy models with various combinations of detection (p) and occupancy (ψ) covariates. In this process, a loop iterates over possible combinations of covariates affecting detection. For each detection covariate set, a nested loop then considers all possible combinations of covariates that could influence occupancy. Each combination of detection (p_formula) and occupancy (Ψ_formula) covariates is consolidated into a single formula, allowing the occupancy model to assess how both probabilities are affected by these factors. This approach allowed us to rapidly evaluate a comprehensive set of models, enabling a more efficient and accurate assessment of important covariate effects on both occupancy and detection probabilities.

To select the best models, we applied the Akaike information criterion corrected for small sample sizes (AICc) following (Burnham and Anderson 2002), prioritizing the most parsimonious models. We considered models to be comparable if they had a ΔAICc < 2.0 and used AICc weights (wi) to identify the most appropriate models describing tapir occupancy within our study areas (MacKenzie et al. 2006; Nichols et al. 2008). To evaluate the predictive performance of our occupancy model, we conducted a cross‐validation analysis, dividing the data into multiple folds and calculating the mean squared error (MSE) for predictions on validation sets, and we assessed overdispersion using the Pearson *X* ^2^ statistic (Semper‐Pascual et al. 2020). If the overdispersion parameter (*ĉ*) was greater than 1, we applied this value to adjust the standard errors of the parameter estimates, accounting for potential overdispersion in the data (MacKenzie and Bailey 2004).

When models with similar AICc were obtained in some study areas, we used model averaging to calculate weighted parameter estimates across the top‐performing models (those with ΔAICc < 2) to account for model uncertainty (Burnham and Anderson 2002). This approach enabled us to estimate occupancy (Ψ) and detection (*p*) probabilities as a weighted average, where models contributing more to the overall likelihood received higher weights. Model averaging provided robust estimates of covariate effects on occupancy and detection probabilities, allowing for a more comprehensive interpretation of tapir habitat preferences while reducing bias associated with model selection uncertainty (Burnham and Anderson 2002).

## 3 Results

A total of 32,000 trap‐days were conducted between 2015 and 2023. We obtained 352 independent detections of lowland tapirs and observed them in 149 of 380 sites, resulting in a naïve occupancy of 0.392 and detectability of 0.46 across all study areas. The model showed good fit, with no evidence of lack of fit or overdispersion (*p* < 0.034, *ĉ* = 0.0577). The average MSE across all cross‐validation folds was 0.213, reflecting a relatively low prediction error. This implies that the model provides accurate predictions of lowland tapir occupancy.

**FIGURE 2:**
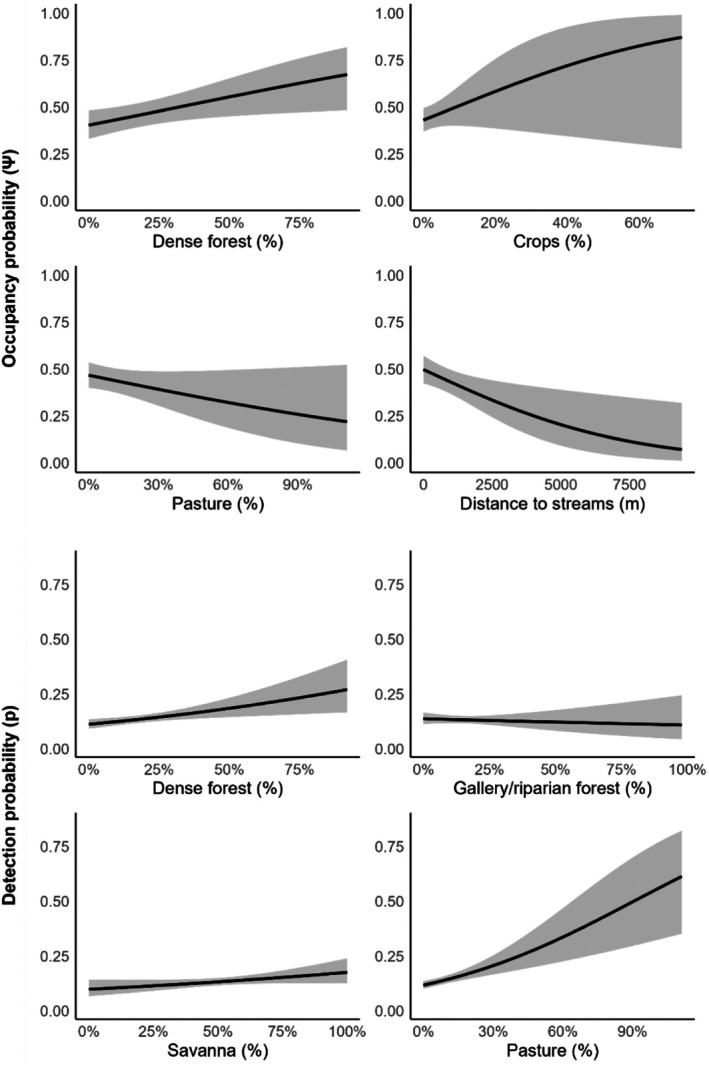
Modeled effects on occupancy (Ψ) and detectability (p) from the top‐ranked single‐season occupancy model. Partial‐dependence plots show predicted Ψ across dense forest cover (%), crop cover (%), pasture cover (%), and distance to streams (m); and predicted p across dense forest cover (%), gallery/riparian forest cover (%), savanna cover (%), and pasture cover (%). Solid lines are model predictions; shaded bands are 95% confidence intervals. All other covariates were held at their standardized means (*z* = 0).

From all possible occupancy models produced in each of the study areas (~3000–10,000), the top predictors of occupancy included: (1) dense forest (forest_d), which had a positive relationship (*β* = 0.334 ± 0.141), indicating that this habitat increased the probability of lowland tapir occupancy (Ψ = 0.58, CI: 0.39–0.64); (2) crops, which also had a positive effect (*β* = 0.232 ± 0.197), suggesting that crop areas may increase occupancy probabilities (Ψ = 0.58, CI: 0.47–0.67); (3) pasture (past), which negatively affected occupancy (*β* = −0.100 ± 0.130), reducing the probability of the species being present (Ψ = 0.45, CI: 0.40–0.51); and (4) distance to streams (d_streams), which showed a negative effect (*β* = −0.354 ± 0.143), with occupancy probability decreasing as the distance from streams increased (Ψ = 0.41, CI: 0.34–0.48). Detectability was most strongly influenced by (1) dense (*p* = 0.57, CI: 0.52–0.62) gallery and riparian forests (forest_g; *p* = 0.49, CI: 0.43–0.54), which all had a positive effect, indicating that these habitats enhance detection probability; (2) savannas (sav), which also had a positive effect, suggesting higher detection likelihood in these open areas (*p* = 0.55, CI: 0.49–0.60); and (3) pastures (past), which similarly increased detectability, suggesting that detection was higher in habitats associated with pastures (*p* = 0.60, CI: 0.55–0.64) (Figure 2, Table 2).

**TABLE 2:** Occupancy (Ψ) and detectability models (*p*) for the lowland tapir in the Orinoquia region of Colombia. These models were analyzed using 4‐day sampling intervals. The table presents the Akaike Information Criterion corrected for small sample sizes (AICc), differences in AICc (∆AICc), Akaike weights (wAICc), and the number of parameters (*K*) for each model. Only models with a ∆AICc < 2 are shown.

Site	Model	AICc	∆AICc	wAICc	*K*
Orinoquia	*p*(dense+gallery+sav + past)Ψ(dense+crops+past+d_streams)	2389.30	0	0.008	10
	*p*(dense+past)Ψ(dense+crops+past+d_streams)	2389.61	0.31	0.007	8
	*p*(dense+gallery+sav + past)Ψ(dense+crops+d_streams)	2389.82	0.51	0.006	9
	*p*(dense+past)Ψ(dense+sav + crops+d_streams)	2389.87	0.57	0.006	8
	*p*(dense+past)Ψ(dense+crops+d_streams)	2390.05	0.75	0.005	7
	*p*(dense+gallery+sav + past)Ψ(dense+crops+d_streams+d_crops)	2390.19	0.89	0.005	10
	*p*(dense+past)Ψ(dense+past+d_streams)	2390.26	0.96	0.005	7
	*p*(dense+gallery+sav + past)Ψ(dense+crops+past+d_streams+d_crops)	2390.30	1.00	0.005	11
	*p*(dense+gallery+past)Ψ(dense+crops+past+d_streams)	2390.32	1.01	0.005	9
	*p*(dense+past)Ψ(dense+sav + crops+d_streams+d_crops)	2390.67	1.37	0.004	9
	*p*(dense+gallery+past)Ψ(dense+past+d_streams)	2390.80	1.50	0.004	8
	*p*(dense+gallery+past)Ψ(dense+crops+d_streams)	2390.83	1.53	0.004	8
	*p*(dense+past)Ψ(dense+crops+d_streams+d_crops)	2390.89	1.59	0.003	8
	*p*(dense+gallery+sav + past)Ψ(dense+past+d_streams)	2390.92	1.62	0.003	9
	*p*(dense+gallery+sav + past)Ψ(dense+open+crops+past+d_streams)	2390.94	1.63	0.003	11
	*p*(dense+gallery+sav + past)Ψ(dense+sav + crops+d_streams)	2390.97	1.67	0.003	10
	*p*(dense+past)Ψ(dense+crops+past+d_streams+d_crops)	2390.99	1.69	0.003	9
	*p*(dense+past)Ψ(dense+d_streams)	2391.03	1.73	0.003	6
	*p*(dense+past)Ψ(dense+gallery+crops+past+d_streams)	2391.21	1.91	0.003	9
	*p*(dense+gallery+past)Ψ(dense+sav + crops+d_streams)	2391.22	1.92	0.003	9
	*p*(dense+past)Ψ(dense+sav + crops+past+d_streams)	2391.27	1.97	0.003	9
	*p*(dense+gallery+open+sav + past)Ψ(dense+crops+past+d_streams)	2391.29	1.98	0.003	11

**FIGURE 3:**
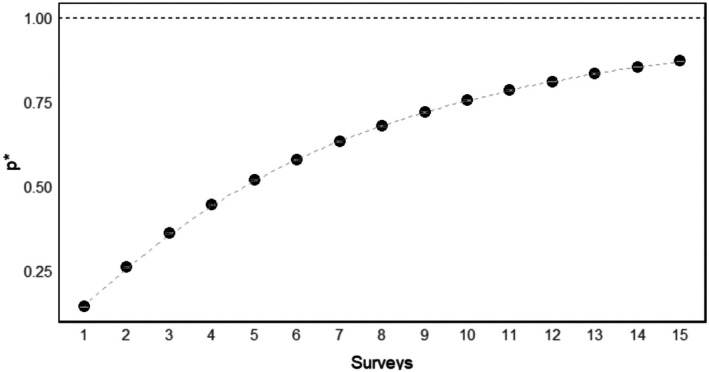
Probability of detecting at least one individual tapir based on a sampling period of 15 surveys (60 days). Each point is the estimated probability (p*) of detecting at least one tapir for a given number of surveys. The 95% confidence intervals indicate that the estimated detection probabilities are precise.

The probability of detecting at least one individual (p*) increased as the number of surveys increased. In our study, the probability increased rapidly from lower values as the number of surveys increased and showed a near asymptotic behavior at about 12–15 surveys, where p* approached values close to 1 (Figure 3). It is important to note that each survey is composed of a 4‐day sampling interval, meaning that 15 surveys represent a total of 60 days of sampling effort. This indicates that a higher number of surveys significantly increases the probability of detecting at least one individual tapir.

**FIGURE 4:**
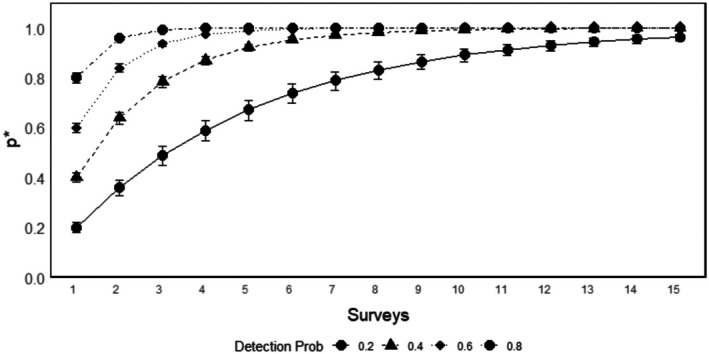
Probability of detecting at least one lowland tapir (p*) as a function of the number of surveys conducted, shown for different baseline detection probabilities (0.2, 0.4, 0.6, and 0.8). Error bars represent the 95% confidence intervals.

In the multiple scenario analysis (Figure 4), p* demonstrated a clear dependence on detection probability. The probability of detecting at least one individual, p*, increases as the number of surveys rises and approaches a value near 1.0 for higher detection probabilities. Scenarios with higher detection probabilities (i.e., 0.6 or 0.8) reach this maximum value with fewer surveys, whereas lower detection probabilities (i.e., 0.2 or 0.4) require considerably more surveys to achieve a similar p* level. These results emphasize the combined influence of the number of surveys and baseline detection probability on the likelihood of detecting the species, underscoring the importance of incorporating both factors into effective survey design.

**FIGURE 5:**
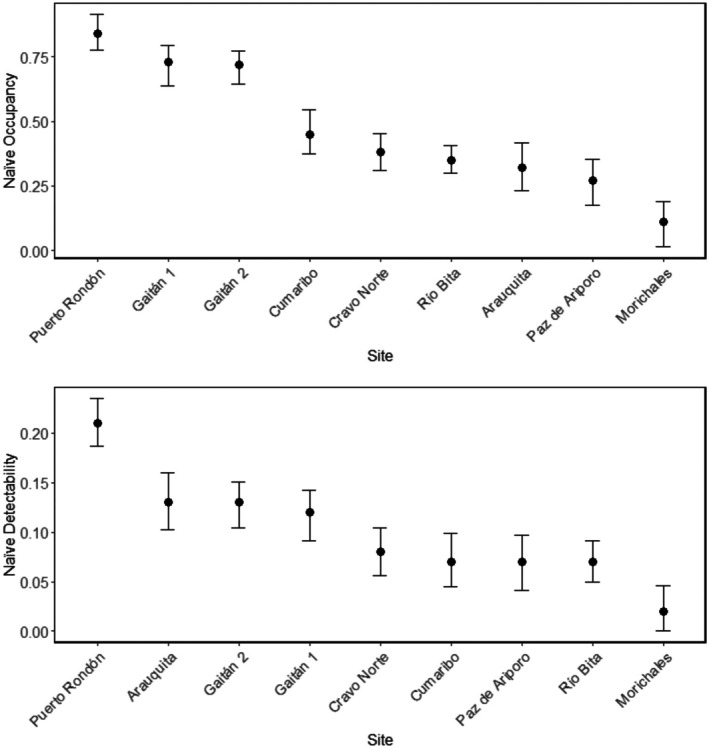
Naïve occupancy and detectability of the lowland tapir across study sites in the Colombian Orinoquia. Sites are arranged from left to right according to decreasing naïve occupancy and detectability. Puerto Rondón shows the highest naïve occupancy and detectability, while Morichales exhibits the lowest. Error bars represent the standard error of the naïve occupancy estimate for each site, reflecting the variability and uncertainty in detection.

The regions with the highest occupancy rates and detectability for lowland tapirs were Puerto Rondón‐Arauca (Ψ = 0.01–0.98, *p* = 0.19–0.31) and Puerto Gaitán‐Meta (Ψ = 0.10–0.95, *p* = 0.10–0.16). In contrast, the areas with the lowest occupancy and detectability rates were Paz de Ariporo (Ψ = 0.13–0.51, *p* = 0.07–0.34) and Morichales (Ψ = 0.09, *p* = 0.09–0.19) in Casanare (Table 3, Figure 5). The occupancy covariates that consistently influenced tapir presence across the study areas included dense forest, gallery and riparian forests, crops, and distance to roads, while the most variable covariates were savannas, pasture, and water‐related variables (rivers, streams, and distance to rivers). Similarly, detectability was consistently explained by gallery and riparian forests as well as water‐related variables, whereas the most variable covariates were dense forest, pasture, and crops.

**TABLE 3:** Occupancy (Ψ) and detectability models (*p*) constructed for the lowland tapir in multiple sites within the Departments of Arauca (Puerto Rondón, Arauquita, Cravo Norte), Casanare (Paz de Ariporo, Morichales), Vichada (Bita, Cumaribo), and Meta (Gaitán), based on camera trap data collected between 2015 and 2023.

Site	Model	AICc	∆AICc	wAICc	*K*
Puerto Rondón	*p*(dense) Ψ(dense+roads)	249.6222	0	0.0702	5
	*p*(dense) Ψ(sav + roads)	249.6281	0.0059	0.0699	5
	*p*(river) Ψ(dense+roads)	249.8782	0.2560	0.0617	5
	*p*(river) Ψ(sav + roads)	249.8858	0.2636	0.0615	5
Arauquita	*p*(dense+open+secondary+past) Ψ(open+fires)	123.9251	0	0.0186	8
	*p*(dense+open+secondary+past) Ψ(past+fires)	123.9292	0.0041	0.0186	8
	*p*(dense+open+secondary+past) Ψ(fires)	124.2076	0.2825	0.0162	7
	*p*(dense+open) Ψ(sav)	125.3065	1.3814	0.0093	5
Cravo Norte	*p*(gallery+d_crops) Ψ(gallery+crops)	419.4487	0	0.0099	6
	*p*(d_crops) Ψ(gallery+crops)	420.2769	0.8283	0.0066	5
	*p*(gallery+d_crops) Ψ(gallery+crops+d_crops)	420.4679	1.0193	0.0060	7
	*p*(gallery+d_crops) Ψ(gallery+open+crops)	420.5988	1.1502	0.0056	7
Paz de Ariporo	*p*(past) Ψ(d_crops)	128.8302	0	0.0116	4
	*p*(gallery) Ψ(d_crops)	129.3959	0.5658	0.0087	4
	*p*(past) Ψ(gallery)	129.8203	0.9902	0.0071	4
	*p*(past) Ψ(sav)	129.8209	0.9908	0.0070	4
Morichales	*p*(dense) Ψ(crops+d_rivers)	58.93519	0	0.0394	5
	*p*(gallery) Ψ(crops+d_rivers)	59.83155	0.8964	0.0252	5
	*p*(dense+gallery) Ψ(crops+d_rivers)	61.36152	2.4263	0.0117	6
	*p*(secundary) Ψ(crops+d_rivers)	61.40917	2.4740	0.0114	5
Río Bita	*p*(gallery+d_water+d_prot) Ψ(gallery+d_prot)	394.9394	0	0.0533	7
	*p*(gallery+d_water+d_prot) Ψ(gallery)	397.072	2.1326	0.0183	6
	*p*(gallery+d_water+d_prot) Ψ(gallery+d_water+d_prot)	397.0818	2.1424	0.0182	8
	*p*(dense+gallery+d_water+d_prot) Ψ(gallery+d_prot)	397.1214	2.1820	0.0179	8
Cumaribo	*p*(past) Ψ(sav + d_streams+d_roads)	249.4572	0	0.0145	6
	*p*(past) Ψ(sav + d_rivers+d_roads)	249.7417	0.2845	0.0126	6
	*p*(past) Ψ(sav + d_roads)	250.0571	0.5999	0.0107	5
	*p*(d_roads) Ψ(sav + d_rivers+d_streams+d_roads)	250.7351	1.2779	0.0077	7
Gaitán 1	*p*(water) Ψ(sav + d_crops+d_roads)	426.0304	0	0.0639	6
	*p*(water) Ψ(dense+d_crops+d_roads)	427.0483	1.0179	0.0384	6
	*p*(water) Ψ(dense+sav + past+d_crops+d_roads)	427.3275	1.2971	0.0334	8
	*p*(water) Ψ(dense+sav + d_crops+d_roads)	427.3344	1.3040	0.0333	7
Gaitán 2	*p*(water) Ψ(gallery+sav + d_crops+d_roads)	424.3154	0	0.0870	7
	*p*(water) Ψ(sav + d_crops+d_roads)	426.0304	1.7151	0.0369	6
	*p*(gallery+sav + water) Ψ(gallery+sav + d_crops+d_roads)	426.3769	2.0615	0.0310	9

## 4 Discussion

This study represents the most extensive and comprehensive efforts to evaluate lowland tapir populations, encompassing 9 years of fieldwork, over 32,000 trap‐days, and nearly 400 sampling sites. The breadth and depth of this dataset significantly enhance our understanding of tapir ecology in one of the most rapidly changing landscapes of South America. By systematically integrating occupancy modeling, habitat assessments, and anthropogenic impact analyses, our findings not only offer robust, data‐driven insights into the species' habitat requirements and behavioral patterns but also provide an empirical foundation for guiding conservation actions.

In general, tapir occupancy and detectability were relatively low (naïve Ψ = 0.40; region‐wide *p* = 0.46), suggesting that the species is both rare and difficult to detect across much of the Orinoquia region. Our models revealed that occupancy was significantly higher in undisturbed habitats like continuous forests (Ψ ~ 0.55–0.65) than in human‐modified landscapes such as pastures (Ψ ~ 0.20–0.30). This directly supports our first hypothesis that occupancy increases with dense forest cover and decreases in pasture‐dominated areas. Occupancy also increased with proximity to streams, consistent with our second hypothesis. However, contrary to expectations, gallery/riparian forests were not strong predictors of occupancy, and crop cover showed a positive rather than negative effect. This contrast highlights that while some hypotheses were supported, others were only partially confirmed, underscoring the importance of testing assumptions in the Orinoquia context rather than extrapolating solely from other regions.

The strong, positive effect of distance to road on occupancy (*β* = 0.72 ± 0.24) further indicates that tapirs are sensitive to human infrastructure. These low probabilities are consistent with the species' biology, naturally low population densities due to slow reproductive rates and large body sizes (Medici 2010; Varela et al. 2019) but are exacerbated in human‐modified areas. This pattern indicates that habitat loss, particularly of dense forests, is a stronger driver than fragmentation per se, although both processes interact. This aligns with several studies that also reported low‐to‐moderate occupancy in fragmented or non‐protected landscapes, including Bolivia (Ψ = 0.39; Wallace et al. 2020), the Brazilian Pantanal (Ψ = 0.57; Regolin et al. 2021), and Argentina (Ψ = 0.40; Cruz et al. 2014; Ψ = 0.33; Bardavid et al. 2024). In contrast, considerably higher occupancy probabilities have been reported from extensive protected areas in the Atlantic Forest of Brazil (Ψ = 0.77; Ferreguetti et al. 2017).

Our analysis pooled data across a 9 year study period to estimate average occupancy probability during this window. Because our sampling design was spatially extensive but not a fixed temporal sampling, we treated each fieldwork period as a single‐season snapshot and extracted year‐specific land‐cover data so that landscape dynamics are represented in the covariates rather than through explicit colonization or extinction modeling. While the landscapes we sampled are indeed dynamic, this approach provides a robust, integrated measure of tapir occurrence across multiple sites and habitat conditions over a much longer timeframe than most previous studies. This offers a more comprehensive baseline for understanding tapir persistence in heterogeneous and changing environments.

Furthermore, detection probabilities varied significantly by habitat, being lowest in gallery and riparian forests (*p* = 0.49) compared to open savannas (*p* = 0.55) or pasture (*p* = 0.60); dense forest showed slightly higher detection (*p* = 0.57). The similarly low detection in gallery and dense forests is likely due to visual obstruction from complex vegetation, which reduces the camera's effective detection zone. However, the fact that detection was lowest in gallery forests, even below that of dense forests, suggests an additional behavioral component. Tapirs may move more freely and range over larger areas in these continuous forest corridors, lowering the likelihood of repeated captures at any single station. At the same time, gallery forests in human‐modified landscapes are often used for timber extraction and occasional hunting, potentially increasing tapir wariness or nocturnality. Together, these factors imply that low detection in gallery forests reflects both methodological bias and behavioral ecology rather than simply camera placement. Recognizing these habitat‐specific mechanisms is essential for refining monitoring protocols and accurately distinguishing between true abundance and detection probability.

Tapir occupancy increases in areas with dense forest cover and proximity to water resources (i.e., streams and wetlands), consistent with previous research (Ferreguetti et al. 2017; Medici et al. 2022). Interestingly, dense forests appear to have a more significant effect on occupancy than gallery and riparian forests, suggesting that dense forest habitats play a critical role in the conservation of lowland tapirs. While our models did not include a direct measure of fragmentation, the strong positive effect of large dense forest patches and the negative effect of pastureland on occupancy suggest that tapirs in our region are sensitive to habitat subdivision. This inferred sensitivity aligns with the well‐documented negative effects of fragmentation on large mammals, which include reduced habitat connectivity, limited access to essential resources, increased mortality risks, and restricted gene flow between populations (Haddad et al. 2015). For tapirs specifically, studies have demonstrated that fragmentation can lead to reduced genetic diversity and increased population isolation (Medici et al. 2022; Saranholi et al. 2022). While tapirs can use riparian corridors for movement, our results imply these may not fully compensate for the loss of core forest habitat at the landscape scale. Therefore, effective conservation actions should prioritize habitat restoration to improve connectivity between forest patches, which would help ensure tapir populations can maintain genetic diversity and access critical resources.

Our models reveal a complex pattern of tapir habitat use in the Orinoquia. While gallery and riparian forests are the dominant forest types, tapir occupancy was highest in dense forests. This is likely a function of both habitat quality and spatial configuration: dense forests not only provide key resources but also consist of large, contiguous patches, whereas gallery/riparian forests are confined to linear belts. Larger patches provide more interior habitat, reduce edge effects, and can support more stable populations (Fahrig 2003). Likewise, water availability significantly increased both occupancy and detectability, as tapirs use water bodies for multiple purposes such as thermoregulation, evasion of predators (jaguars and pumas), removal of ectoparasites, and foraging (Medici 2010; Oliveira‐Santos et al. 2010). However, tapirs also used other habitats. The relatively high detection probability in savannas is likely due to tapirs traversing the open matrix to access key resource patches, including water. Specifically, resource patches like morichales ( *Mauritia flexuosa* ), which are associated with water and serve as important food sources, and saladillales (*Caraipa llanorum*), which act as stepping stones, facilitate movement across the landscape (Alviz et al. 2023).

The savanna acts as a permeable but likely high‐risk matrix. Tapirs can move through it by using these scattered resources (both morichales and saladillales), but this dependence and the higher visibility likely increase their vulnerability to threats. Therefore, the presence of tapirs in savannas likely represents functional connectivity (movement) rather than high‐quality habitat (reproduction, long‐term survival). Our data strongly support the argument that large, dense forests with access to water are the most critical habitats for sustaining tapir populations. The use of savannas underscores the importance of conserving these key resource patches and water sources to maintain connectivity, allowing tapirs to access their core forest habitats.

Evidence from our study suggests that land‐use change significantly influences tapir detectability. In Puerto Rondón (Arauca), roads had a strong negative effect on occupancy, consistent with tapirs avoiding areas near roads (Ferreguetti et al. 2017). Roads can also provide easier access for human disturbance, including hunting and timber extraction, making them important predictors of tapir avoidance (Semper‐Pascual et al. 2020). Land management practices, such as the use of fire, may affect tapir occupancy, as savanna fires may displace individuals and reduce their presence in transformed areas. Conversely, our models grouped all crops as a single covariate, but the positive effect we detected is most consistent with the presence of small‐ and medium‐scale subsistence plots (e.g., plantain, yucca), as suggested by local reports. Importantly, we did not detect tapirs in areas dominated by large monocultures such as oil palm and rice, so their absence there is discussed qualitatively rather than modeled directly.

Our findings highlight that human–tapir interactions in agricultural landscapes represent a localized but significant conservation concern. For a species with low reproductive rates, even occasional losses from conflicts or road accidents can threaten local population persistence. In our study, these conflicts are most likely to occur at the interface between small‐scale crops and forest edges, underscoring the need for management actions at this boundary. Community‐based monitoring programs involving farmers and residents could provide critical data on tapir visits and crop damage, enabling targeted mitigation such as fence reinforcement or temporary deterrents during harvest seasons. Integrating local knowledge with ecological data on movement and habitat use will improve corridor planning and reduce conflict while maintaining habitat connectivity.

Pastures established for livestock negatively impact tapir occupancy, likely because of the low plant richness and homogeneity of these landscapes, offering fewer foraging opportunities. This association also reflects the broader effects of habitat fragmentation caused by agricultural conversion, rather than fragmentation measured directly in our models. As hypothesized, these findings support the idea that livestock‐driven conversion to pasture reduces habitat suitability for tapirs by both simplifying plant communities and fragmenting forest cover. Our results emphasize the importance of addressing this form of landscape subdivision and managing land use sustainably to mitigate these impacts.

Puerto Rondón (Arauca) and Puerto Gaitán (Meta) exhibited high occupancy probabilities (~0.8), indicating that these regions are crucial for lowland tapir conservation. These sites feature extensive dense forest along the Cravo Norte and Manacacías Rivers, which likely support stable tapir populations. Consequently, protecting these areas from habitat loss, poaching, and land‐use changes should be prioritized in conservation strategies. In contrast, Morichales and Paz de Ariporo in Casanare showed low occupancy probabilities (~0.2), suggesting that tapirs are infrequently recorded and detected in these areas. This may be due to significant environmental pressures, such as the expansion of monocultures, particularly rice crops, and extensive livestock farming. Despite Morichales having the largest concentration of moriche palms in the Orinoquia region, the area has undergone extensive forest and savanna exploitation and transformation. Restoring these forests and savannas could improve habitat suitability for tapirs.

Regarding detectability, higher values observed in areas like Puerto Rondón indicate that tapirs were more frequently detected when present. While detectability (*p*) in occupancy models is distinct from abundance per se, higher encounter rates (e.g., more photos) can indirectly reflect greater local density or more predictable movement patterns, which in turn increase the likelihood of detection during a survey (Ferreguetti et al. 2017; Medici et al. 2022). Conversely, lower detectability in regions such as Arauquita may be explained by both ecological and anthropogenic factors. Here, extensive fragmentation and forest loss, estimated at around 40% over the past decade, are likely to reduce the frequency of tapir use of sampled sites. In addition, increased human activity and hunting pressure may drive more cryptic behavior (Monette et al. 2020), further lowering the chance of camera encounters even when animals are present.

Similarly, in locations such as Cravo Norte (Arauca) and Bita (Vichada), despite the existence of protected areas and ongoing conservation initiatives, both detection and occupancy probabilities remain relatively low. This pattern may not necessarily indicate scarcity but rather may reflect the broader ecological context: in these well‐preserved landscapes, tapirs have access to extensive, continuous habitats, which allow them to disperse more widely instead of being concentrated in smaller patches. As a result, individuals are less likely to be repeatedly recorded at any single camera station, reducing detection probability despite potentially stable populations.

In Cumaribo (Vichada), where land‐use change and livestock pressure are relatively low, dense and riparian forests remain well conserved. However, threats persist from expanding road networks and subsistence hunting by local communities. Although hunting of tapirs is legally prohibited, limited enforcement in remote areas allows occasional takes for cultural or subsistence purposes (Melo et al. 2015; Valsecchi et al. 2014). These findings highlight the need to integrate ecological data with local governance and community‐based monitoring programs that strengthen compliance and awareness.

Our results also point to contrasting management priorities. In areas with low detectability, multi‐method monitoring, combining camera traps with acoustic sensors, could improve detection efficiency, while areas with high occupancy should be prioritized for habitat protection and connectivity initiatives involving local governments and private landholders. Finally, although not directly tested here, projected climate‐driven changes in precipitation and fire regimes (Etter et al. 2011) may reduce forest cover and water availability, exacerbating existing pressures. Because tapir occupancy was strongly linked to dense forests and proximity to streams, future monitoring should incorporate climate projections to anticipate distributional shifts and guide adaptive management for long‐term population resilience.

## Author Contributions

**Ángela Alviz:** conceptualization (lead), data curation (lead), formal analysis (lead), funding acquisition (lead), investigation (lead), methodology (lead), project administration (lead), resources (lead), software (lead), writing – original draft (lead), writing – review and editing (lead). **Karen E. Pérez‐Albarracín:** conceptualization (supporting), funding acquisition (lead), resources (lead), supervision (supporting). **Jorge Salazar‐Bravo:** supervision (supporting), validation (supporting), writing – review and editing (supporting). **Richard D. Stevens:** conceptualization (equal), methodology (supporting), project administration (supporting), supervision (lead), validation (lead), visualization (lead), writing – review and editing (equal).

## Funding

This work was supported by World Wildlife Fund (EF11870).

## Conflicts of Interest

The authors declare no conflicts of interest.

## Data Availability

The data supporting this study are available from the corresponding author upon reasonable request. Due to privacy considerations, raw data cannot be shared publicly. However, all analysis scripts and code used in this study are available on GitHub at https://github.com/aalviz86/TapirOccupancyModeling.git.
